# The Applicability of Behaviour Change in Intervention Programmes Targeted at Ending Female Genital Mutilation in the EU: Integrating Social Cognitive and Community Level Approaches

**DOI:** 10.1155/2013/324362

**Published:** 2013-07-29

**Authors:** Katherine Brown, David Beecham, Hazel Barrett

**Affiliations:** Faculty of Business, Environment and Society, Coventry University, Priory Street, Coventry CV1 5FB, UK

## Abstract

With increased migration, female genital mutilation (FGM) also referred to as female circumcision or female genital cutting is no longer restricted to Africa, the Middle East, and Asia. The European Parliament estimates that up to half a million women living in the EU have been subjected to FGM, with a further 180,000 at risk. Aware of the limited success of campaigns addressing FGM, the World Health Organization recommended a behavioural change approach be implemented in order to end FGM. To date, however, little progress has been made in adopting a behaviour change approach in strategies aimed at ending FGM. Based on research undertaken as part of the EU's Daphne III programme, which researched FGM intervention programmes linked to African communities in the EU (REPLACE), this paper argues that behaviour change has not been implemented due to a lack of understanding relating to the application of the two broad categories of behaviour change approach: individualistic decision-theoretic and community-change game-theoretic approaches, and how they may be integrated to aid our understanding and the development of future intervention strategies. We therefore discuss how these can be integrated and implemented using community-based participatory action research methods with affected communities.

## 1. Introduction

Female genital mutilation (FGM), sometimes called female circumcision or female genital cutting, is a deep rooted traditional practice that adversely affects the health and well-being of millions of girls and women. It is estimated that 100–140 million females worldwide have been subjected to FGM and that 3 million are at risk each year [1]. The practice is common in 28 countries in Africa as well as parts of the Middle East and Asia [1, 2]. However, with increasing international migration, the practice of FGM is no longer restricted to the traditional practising countries. In 2010, the European Parliament estimated that up to half a million women living in Europe had been subjected to FGM, with a further 180,000 at risk [3, 4]. According to the UNCHR, nearly 20,000 women from FGM practising countries applied for asylum to the EU in 2011 with an estimated 8,809 female applicants aged 14–64 likely to be affected by FGM [5]. In addition to those coming to the EU who have already been subjected to FGM, there is anecdotal evidence supported by criminal prosecutions, particularly in France and Sweden, that suggests that FGM is conducted in the EU [6–9]. This has led to the implementation of FGM elimination campaigns in the EU. 

There are a growing number of studies which demonstrate a significant association between FGM and various gynaecological and pregnancy complications. World Health Organization (WHO) reports [10, 11] conclude that FGM has negative implications for women's health, with women who have undergone FGM more likely than others to have adverse obstetric outcomes. FGM has no health benefits and harms girls and women both physically and mentally. These impacts occur at the time of the procedure as well at adulthood, particularly motherhood. All forms of FGM have psychological effects, particularly related to female sexuality and sexual relationships. The UN regards FGM as a violation of female reproductive rights [12, 13], and thus the ending of FGM is of relevance to all health professionals. Understanding the issues associated with preventing FGM is particularly relevant to health professionals who work with FGM affected and at risk women and girls, since they are in a position to communicate directly with affected community members and may also be linked with organisations which engage in prevention as well as obstetric and gynaecological treatment of FGM complications.

The WHO, United Nations (UN), Unicef, and other anti-FGM organisations have adopted various strategies in order to raise awareness and work towards ending FGM. Such efforts have centred around four main approaches. These include bodily and sexual integrity; human rights; legislative; and the health approach. Thirty years on since the WHO called for the ending of FGM, there is conflicting evidence as to whether these approaches have led to a reduction in the practice [14, 15]. In 1999, aware of the limited success to date in eliminating FGM, the WHO recommended a behavioural change approach be implemented in order to move closer to the elimination of FGM [16]. In 2002, the Frontiers in Reproductive Health and Population Council (FRHPC) produced a review of FGM interventions and called for research to be informed by behaviour change theory (BCT) [17]. They suggested that few evaluations of interventions assess their impact on important outcomes including “knowledge, beliefs, attitudes, and behaviors” concerning FGM and that BCT is needed to establish how interventions work [17, page 1]. Despite the numerous calls for more targeted behaviour change approaches to the issue of FGM, little progress has been made in implementing and/or evaluating behaviour change approaches [2].

This paper is based on research undertaken as part of the EU's Daphne III programme, which researched FGM intervention programmes linked to African communities in the EU (REPLACE). One of the aims of this 12-month project was to work with FGM affected communities and nongovernmental organisations implementing FGM elimination interventions to understand the barriers to the ending of FGM and to assess the appropriateness of NGO intervention materials and awareness raising activities. The project used a community-based participatory action research approach to try to understand why FGM intervention programmes have not delivered an end to FGM in the EU. The results of this part of the research were then applied to a grounded health behaviour change approach in line with WHO's [16, page 2] call for the reorientation of anti-FGM communication strategies “from awareness raising to behaviour-change intervention approaches”. REPLACE produced a toolkit designed to introduce behaviour change approaches to those working to end FGM amongst affected communities in the EU [18]. This was achieved by integrating social cognitive and community level behaviour change intervention strategies. 

In this paper we argue that because of the social aspects characteristically associated with FGM, including gender norms, power relations, and the level of social capital associated with the practice, it is fundamentally important that behaviour change approaches adopt a holistic approach, rather than focusing on the individual or group dynamics of attitude and behaviour change. We essentially argue that behaviour change approaches cannot only focus on the individual and thus neglect the wider social dynamics nor can community based approaches, such as social convention theory [19], overlook intrapersonal and interpersonal aspects located at the individual level. In order to provide context for arguing for the applicability of a more holistic behaviour change approach, we introduce the four traditional approaches to ending FGM. 

## 2. The Four Traditional Approaches to Ending FGM

The REPLACE project used community-based participatory action research (PAR) methods to work with FGM affected communities in the Netherlands and the UK, as well as established nongovernmental organisations working to end FGM amongst these communities, in order to understand the current barriers to the ending of FGM and to assess how these related to the four traditional approaches to ending FGM. PAR was used as it empowered members of FGM affected communities, in this study from the Somali and Sudanese communities, to actively engage in gathering knowledge about individuals' experiences and the personal and community issues preventing them from abandoning the practice. The use of “cultural insiders” to conduct the research was consistent with an essential aspect of PAR; namely, that research is conducted “with” rather than “on” the community. This methodology proved to be an effective way to engage with marginalised and vulnerable groups concerning a practice which is illegal in the EU. It also enabled those involved in the research, including NGOs, to evaluate and reflect on their actions and interventions. 

### 2.1. Bodily and Sexual Integrity Approach

The bodily and sexual integrity approach has been informed by feminist writings concerning women's sexual integrity and pleasure. Johansen [20] has commented that, because Western “second wave feminists” use the clitoris as a symbol of female sexuality, the practice of FGM is seen as the antithesis of women's sexual freedom and expression. However, contradictory views may be held by FGM affected communities regarding the role of FGM in reducing women's sexual pleasure, with some holding the belief that genital cutting makes women sexually accessible [20, 21]. 

Amongst the communities that REPLACE worked with it was very apparent that the control of female sexuality is a major driver in the continuation of the practice, with men and older women such as grandmothers being particularly motivated by this. Those working to end FGM in the EU need to be aware that many members of FGM affected communities are deeply concerned about the sexual liberalism prevalent in many Member States [9, 22]. This was confirmed in the REPLACE project where a large number of those involved in the study perceived the bodily and sexual integrity message as a threat to their deeply held religious and cultural beliefs.

The bodily integrity message emphasises women's individuality regarding sexual pleasure, but, if we accept that sexual enjoyment is shaped, mediated, and controlled through social institutions and understandings of sex and sexuality [18, 23], then we must accept that women's enjoyment of sex will be informed by their own experiences embedded within their specific sociocultural environs. For those women who perceive their sexual enjoyment to be “normal” and providing them a sense of intimacy, then the credibility of this approach may be questioned. Furthermore, women's sexual enjoyment may differ depending on the type of FGM experienced [6, 24]. Many women in the REPLACE study stated that sexual relations caused them physical and psychological pain, a minority reported that they still enjoyed sexual intimacy with their husbands. This is in line with findings from other research conducted in the EU [6, 25, 26] and highlights the contradictory nature of women's sexual experiences and highlights the paradoxical nature of using this approach with FGM affected communities to end the practice. 

Because of the variance in women's sexual experiences and the fact that these messages stem from a “Western” perspective of female sexuality and embodied experience [27, 28], REPLACE found that it did not resonate effectively with affected communities. It has to be recognised that FGM affected communities in the EU may perceive the bodily and sexual integrity message as a threat to their deeply held religious beliefs and conservative values regarding women's sexuality. 

### 2.2. Human Rights Approach

The human rights approach to ending FGM has been influential at an international level, with the UN, the European Parliament, and many governments in the developed world framing FGM as a fundamental violation of the human rights of girls and women. The foundation of this approach is the belief that there are certain universal rights which need to be respected. Some have questioned whether this approach is too “Western centric” in that it is a product of the “Western” liberal democratic tradition, which places emphasis on individual rights rather than “community” rights [29]. Furthermore, some have raised the question of whose “rights” are actually embodied in the UN declaration of human rights [22, 30]. 

For most participants in the REPLACE project the human rights approach to ending FGM was problematic. Issues such as “choice” and consent [31] were discussed at great length with many asking how FGM can be condemed as a human rights violation when male circumcision is not [32] and when the practice of labioplasty is on the increase amongst “Western” woman [33]. Many also highlighted the inconsistency of the human rights approach to ending FGM, in particular questioning the precedence of the right to the security of the person (Article 3) over issues related to religious beliefs (Article 18). Dustin and Phillips [34] point out that the freedom to practice one's religion, freedom from racial discrimination, and the protection of the rights of the child are all in conflict with each other with respect to the issue of FGM. Most communities involved in the REPLACE study questioned the relevance of the human rights approach to ending FGM due to its focus on individual human rights and lack of cultural relevance and sensitivity particularly with reference to religious freedom. It was perceived that Western liberal interpretations of human rights were being imposed on them. As a result a number of respondents suggested that continuing to perform FGM could be interpreted as a means by which communities retain a sense of their “ethnic identity” particularly if they feel they are being discriminated against by the wider society due to their religious beliefs and perceived “right” to perform FGM [20].

Despite the somewhat problematic nature of the human rights approach, most NGOs and governments have adopted this framework to address the issue. It has undoubtedly been politically powerful, with the European Union adopting a “zero tolerance” approach to FGM, meaning that any form of genital cutting is considered a violation of human rights [35]. Nevertheless, REPLACE demonstrated that the messages developed by NGOs and government bodies to tackle FGM need to account for contradictions inherent in the declaration of human rights and associated legislation [34]. Those adopting a human rights framework need to be aware of the wider social and political structures that enable or constrain individuals, particularly women, to affirm their rights and exercise choice. Indeed, it has been argued that, in order to make human rights messages more powerful, they need to address related complexities and ambiguities that confront FGM affected communities residing in the EU; in other words, they need to exemplify the lived realities of individuals and communities in order to be effective [36]. This is exactly the approach REPLACE adopts. 

### 2.3. Legislative Approach

Most EU Member States have legislation which criminalises the practice of FGM, either as a specific criminal act or as an act of bodily harm or injury. Many countries also have an extraterritoriality clause which makes it illegal for their citizens to travel abroad to have FGM performed. A number of Member States' legislation such as the UK's Female Genital Mutilation Act (2003) only applies to those individuals with permanent residency rights. Therefore, people on temporary residency visas, such as students, as well as undocumented migrants and asylum seekers, are not bound by the legislation.

Even though legislation has been in place for a number of years, there have been few FGM related convictions in the EU. It has been argued that there is a general reticence about enforcing the legislation within the UK, with Phillips [37] arguing that the UK Female Genital Mutilation Act (2003) is simply a “symbolic piece of legislation…designed to point a finger of blame at particular cultural communities than to eradicate harms to women” (page 129). There are conflicting data regarding the number of criminal court cases related to FGM across Europe, with Nijboer et al. [8] reporting 42 documented criminal cases relating to FGM in France and the European Institute for Gender Equality noting that there has been 41 cases across 6 Member States relating to FGM [2]. This discrepancy notwithstanding, there is some debate as to whether legislation acts as a deterrent [8] or whether specific criminal law provisions, such as those in the UK, are more effective in prosecuting and punishing FGM [2]. Nijboer et al. [8] argue that parents and excisors simply become more aware of the legislation and find ways to thwart it; for example, by going to another EU member state where it is believed there is less risk of prosecution, such as the UK. There is evidence to suggest that, because of the lack of prosecutions, particularly in the UK, there is a belief amongst some communities that, because the law has not been enforced, it is unlikely that individuals are going to be prosecuted [6, 38].

What became evident in the research undertaken in the REPLACE project was a lack of understanding of the law related to FGM. Many respondents were under the misapprehension that FGM legislation was only applicable to type III FGM (infibulation) which was recognised by the majority of people involved in the study as a violation of human rights and detrimental to the health of girls and women. Most condemned it as a practice. For the most part, however, the less invasive types of FGM, often referred to as “sunna”, were not regarded as “mutilation”, and therefore few understood that these forms of FGM are also against the law. 

Although it is questionable as to whether legislation alone promotes behaviour change, it does provide an “enabling environment” for both anti-FGM campaigners and community members who have taken the decision to abandon the practice [2]. Legislation therefore provides a structural framework within which campaigners and individuals can reject arguments that promote the continuation of FGM [39]. However, there is a danger that criminalisation drives FGM further “underground”, with inexperienced circumcisers conducting the practice [39] or girls and women not seeking health care for complications arising from FGM due to a fear of prosecution [14].

### 2.4. The Health Approach

Anti-FGM campaigners and international organisations, such as the UN, WHO, and Unicef began to emphasise the negative health consequences of FGM in the early 1980s. Boyle [40] argued that this approach arose out of an agreement between feminist activists and international organisations, that such an approach would be considered “apolitical” and not perceived by affected communities as the imposition of “Western” views and ideologies. This approach focuses on the immediate and long-term health consequences of FGM and the irreversibility of the procedure [16, 22]. 

Many African women's groups have adopted the health approach in “sensitization” or “sensibilisation” workshops and national campaigns aimed at ending FGM [41]. In their use of it, campaigners have been reluctant to make a clear distinction between the health consequences of less severe forms of FGM such as “clitoral pricking” (Type IV) and “sunna” (Type I/II) and more severe forms, such as infibulation (Type III) [13, 28]. To differentiate between the types of FGM on the basis that one type poses less of a health risk than another might be seen as condoning “milder” forms of the practice and thus undermines their efforts to eliminate all forms of FGM [42]. 

An unintended consequence of the lack of distinction between FGM types in campaigning has led to more invasive forms of genital cutting being viewed by communities as the only type responsible for negative health outcomes [22, 27, 28]. This finding was confirmed by the REPLACE project. It is not unreasonable to assume that these health messages, particularly highlighting the severity of infibulation, has led to an increase in the “medicalization” of FGM, especially less invasive forms [43]. For example, WHO reports [44] an increase in the number of parents seeking out medical practitioners to carry out the procedure. The REPLACE project found communities accepting the health messages concerning infibulation but not able to relate these messages to other types of FGM. Many queried the stated health complications of “sunna”. It was evident that health messages needed to be more specific and targeted at the various types of FGM. It was also found that some individuals and communities involved in the REPLACE project believed that there are health “benefits” to FGM, in which it improves hygiene or “cleanliness” [18, 45]. These arguments are closely related to religious beliefs about “purity” and spiritual cleanliness and thus are difficult to address via a preventive health message. As Berg et al. [46] suggest, beliefs regarding the continuation of FGM exist at multiple levels, and the contradictory nature of some beliefs need to be accounted for in messages aiming to achieve change. For health messages to be effective they need to accurately represent the lived realities of women who have experienced different forms of FGM; otherwise this will lead to what Shell-Duncan et al. [39] call the “credibility gap”. 

It is difficult to assess the efficacy of these four traditional approaches to ending FGM as few studies have evaluated their success in terms of attitudinal or behaviour change [36]. In addition, without accurate prevalence figures relating to FGM within the EU, it is difficult to measure the success of any of the work aimed at ending FGM to date. The growing numbers of people from FGM affected communities speaking out against the practice are perhaps an indication that there has been some success, but the number of criminal court proceedings highlighted by the EU [2] and anecdotal evidence [6, 18] suggests that FGM continues in an EU context even though it is outlawed. 

As part of the REPLACE project, awareness raising information and activities undertaken by anti-FGM campaigners were reviewed. REPLACE found that anti-FGM programmes could in the main be classed as traditional information, education, and communication. Most focussed on the health approach (with some links to bodily and sexual integrity) and the twinned approaches of human rights and the law. Whilst all materials had accurate and relevant information, only a minority attempted behavioural change communication. When there was a focus on behaviour change communication, it very much emphasised the role of the individual, with little if any acknowledgement of community belief systems, and thus was unlikely to change behaviour. The PAR findings showed that there were often dichotomies in the way individuals and groups of individuals received the information disseminated by anti-FGM campaigners, with many campaigners “delivering” information rather than “listening to” and responding to the specific belief systems of the communities in which they were working.

It was clear to the REPLACE team that intervention campaigners needed guidance on how to incorporate behaviour change intervention into their programmes. The findings of the REPLACE project thus indicated that campaigners and activists needed to engage with communities in order to develop context specific messages and strategies that target emotive and rational cognitive processes that inform attitudinal and behaviour change. This can only be done by adopting a community-based participatory action research methodology. 

Interventionists also need to have clear measures by which they assess the success of an intervention in terms of attitudinal and behaviour change. Denison et al. [47] have suggested that Ajzen's theory of planned behaviour (TPB) [48] could be highly applicable to the issue of FGM. Undoubtedly, TPB can provide a contribution to our understanding of rational or reflective cognitive process of behaviour, but one also has to take account of the emotional impulses that arise from associative learning and/or innate disposition. Below we outline a behaviour change approach which takes into account the macro and community level structures and the interpersonal and intrapersonal factors that enable and/or constrain behaviour change concerning the issue of FGM. 

## 3. Behaviour Change: The Call for a New Approach

A review of the application of behaviour change approaches within an African context and Europe undertaken by Leye [36] illustrated a lack of agreement as to which approach or approaches were most relevant to the issue of FGM. Arguably, this lack of agreement stems from the fact that behaviour change approaches broadly fall into two categories that we describe as (1) theories which focus on individual behaviour change and (2) those which concentrate on how change occurs at a community level. Shell-Duncan et al. [15] refer to these as (1) decision-theoretic models and (2) game-theoretic models. Decision-theoretic models tend to address the rational, reflective, and systematic cognitive processes that individuals engage in when deciding to act. Shell-Duncan et al. [15] identify that many messages used in anti-FGM campaigns to date have applied this “rational” approach when highlighting, for example, the health risks of FGM and the benefits of remaining uncut. Shell-Duncan et al. [15] criticise the “rational” decision-theoretic models as simplistic cost-benefit analyses and propose game-theoretic approaches, such as Mackie and LeJeune's [19] and social convention theory, as being preferable for understanding behaviour change in relation to FGM.

In describing decision-theoretic models as simplistic cost-benefit analyses, we argue that Shell-Duncan and colleagues [15] are misrepresenting the potential of such theories for contributing to our understanding of the continuation of FGM and for intervening more successfully to promote change. Further illustration of their oversimplification of individualistic or decision-theoretic approaches is provided in their [39] application of the stages of change or transtheoretical model (TTM) [49] to FGM. Although Shell-Duncan et al. [39] consider some wider decision-theoretic behaviour change approaches, the work focuses on the stages of change and decisional balance elements of the TTM alone. Self-efficacy and processes of change, other elements of the TTM, are not considered as potentially relevant. Within their concluding remarks, Shell-Duncan et al. [39] conceded that the issue of behaviour change, with respect to the practice of FGM, “remains poorly understood” (page 130). 

We suggest one of the poorly understood aspects of individualistic decision-theoretic theories in this context is that, despite being conceived around psychological processes and their relationship with behaviour or behaviour change within individuals, they are tested on population samples and useful for population-level interventions. We argue that, in order for behavioural change approaches to be more successfully applied in attempts to end FGM, a more coherent and comprehensive understanding of how individualistic decision-theoretic and community level game-theoretic approaches might be integrated, is required. Indeed, this point is noted by Denison et al. [47] when they state that, in order to achieve successful behaviour change, efforts need to be intensified at all levels, which include the individual and group level and community level interventions. In an attempt to move towards achieving this with regards to ending FGM we firstly outline three major game-theoretic or community change models that have been or could be applied to understanding change in FGM practices and some of their strengths and limitations. We then present an example of how concepts from individual (decision-theoretic) and community (game-theoretic) behaviour changes might be synthesised to address the identified limitations.

### 3.1. Game-Theoretic Approaches, Community Change: Social Convention Theory

Social Convention Theory has been applied to understand harmful traditions and cultural practices, such as foot binding amongst Chinese communities and FGM [19]. Mackie and Le Jeune [19] highlight the wider inequalities in society that perpetuate such practices and how aspects such as gender, class, and the desire to improve one's access to social and economic resources may contribute to the establishment and continuation of the practice. To illustrate, in many FGM affected communities, women who have been cut are considered to have maintained their virginity which is desirable for marriage. Consequently, the convention of cutting females' genitals becomes accepted as a social norm as no family wants to suffer the stigma associated with having a daughter considered “unfit” for marriage. The practice of FGM is embedded and reenforced because decisions made about performing FGM are interdependent on decisions made by other intramarrying families in the communities around them; namely they will have their daughters cut in order to improve their likelihood of securing a good marriage partner. In order to end such a social convention it is argued [19] that a critical mass of families within a community must publically renounce the practice; as it is only when communities desist that, individual families will believe it is acceptable and not detrimental to their status not to cut their daughters. This logic underpins Tostan's community intervention programme, which culminates in a community visibly and collectively declaring their renunciation of FGM [50]. 

Abandonment of FGM based on Social Convention Theory is said to be achieved through organised diffusion, involving participants sharing information, persuasion, and debate spread through existing familial and social networks [19]. It is proposed that an entire community need not be persuaded; rather what is required is a motivated critical mass of people to collectively decide that they are willing to abandon the practice. This critical mass need to persuade others to commit to the idea until there are enough (at the tipping point) to act together to make a public commitment to abandon the practice. 

### 3.2. Diffusion of Innovation Theory

Diffusion of Innovation theory [51] arguably offers further theoretical insight into the mechanisms by which one might establish change at a community level. An innovation, in this context, can be any idea, practice, or product that is new to an individual, organization, or population [52]. Diffusion theory describes the characteristics of the people who might adopt an innovation and characteristics of the innovation itself as relevant to individual decision-making and broader adoption in a population [51]. Adopters are classified as innovators, early adopters, early majority, later majority, and laggards, depending on their relevant point of uptake of an innovation. These categories of adopters have been found to have different characteristics (e.g., innovators are venturesome while the late majority are sceptical) that determine their desire to engage with a new innovation and communicate with others about it [53]. If we view the idea of discontinuing the practice of FGM as an innovation, we might describe the people in Mackie and Le Jeune's [19] “critical mass” as innovators and early adopters who may need to engage the early majority in order to reach “the tipping point” for change. What is problematic in terms of ending FGM is determining the characteristics and motivation to ending FGM that typify innovators and early adopters and how can such families be identified and supported.

Abandoning the practice of FGM is likely to be considered as having at least some disadvantages, such as its incompatibly with current behaviour; its complexity; the potential negative impact on social relations; and the potential risks and uncertainty. Consequently, given these likely perceptions, the idea of not practicing FGM is likely to be difficult to diffuse [53]. In addition, Wejnert [54] draws attention to “environmental” factors that affect diffusion including political context, local culture, and increasing levels of globalisation in particular communications and media [54]. In line with Mackie and Le Jeune's [19] argument about the influence of patriarchal society, religion, and culture on the practice of FGM, no one factor is likely to be the direct reason for continuation or discontinuation of the practice (or the spread of innovation), but they are all influential and an important consideration in planning and organising change.

### 3.3. Community Readiness Model

A third model, the Community Readiness Model, developed by Edwards et al. [55] is also relevant for understanding how those interested in working to end harmful practices such as FGM might aim to bring change at a community level and design targeted and effective interventions. This theory, developed through extensive empirical work on programmes to address drug addiction and domestic violence, proposes nine stages of community readiness shown in Table 1.

In order to apply these stages to identified community problems, Edwards et al. [55] have devised methods for assisting in classification of a community. These include the use of key informants who are nonspecialist community members knowledgeable about the issue under investigation in their community. They also describe methods for applying the approach; these include teaching the theory to community members and letting them devise their own strategies and policies designed to move the community through the stages of readiness. Such influential members of the community may well have the characteristics and motivation to become “innovators” or “early adopters” as they would be known under Diffusion of Innovation Theory. Over the course of the community readiness development, general strategies have been devised for moving communities from each stage to the next, and these strategies have been shared as suggestions with communities who have then developed and adapted these to meet their own needs as appropriate within their community context [55]. 

### 3.4. Contribution and Limitations of Community Level (Game-Theoretic) Approaches

Community change approaches such as those outlined above place important emphasis on promoting and facilitating change from within the community. Furthermore, they highlight the importance of challenging the structural constraints that prevent change, for example, promoting positive and supportive environments in which sensitive topics like FGM and sexuality can be discussed [36]. Challenging the material and social constraints preventing abandonment of FGM is embodied in the Tostan programme, which is grounded in Social Convention Theory [50].

Undoubtedly, the Tostan project has made a positive contribution, although Obiora [50] warns us not to perceive a public renouncement of FGM as signalling the elimination of the practice or as the cause of a collective shift. Obiora [50] suggests that the power of cultural and social norms over the individual should not be underestimated, as adherence to these can take precedence over personal intuitions and recognition that continuing the procedure has potential health implications. Indeed, Diop and Askew [56] in their evaluation of NGO intervention strategies in Senegal, Burkina Faso, and Mali report that several traditional practitioners, who underwent “sensitization” programmes and made a statement pledging to abandon FGM, continued the practice. One of the reasons they gave for continuing the practice was that “they were not convinced that what they were doing was wrong” [56, page 134]; this finding supports Obiora's [50] scepticism of public statements renouncing FGM as signifying success. But, more importantly, it highlights the need to construct effective messages that will address the deeply held beliefs of a particular community. If programme developers do not consider how the content of programmes, activities, campaigns, and messages are understood and responded to by individuals (and groups of individuals), then the content and nature may be ineffective or less effective than it could otherwise be. 

Mackie and Le Jeune [19] acknowledge that beliefs and norms are held and understood at the individual level as well as across groups of people and are equally important to the change process. Similarly, those applying diffusion of innovation to health-relevant issues have noted that “potential adopters' perceptions of what the innovation is like”, also need to be taken into consideration [53, page 110]. Thus, beliefs held at the individual level and group level can act as a barrier or can facilitate change. No matter how much communication innovators have with potential early adopters or early adopters have with the “critical mass”, if beliefs about an innovation remain negative and unchanged, adoption may not occur.

Social Convention Theory provides insight into why FGM may have become embedded into communities and also presents a general approach for understanding how communities might organise themselves to change. Additionally, research has found empirical support for the applicability of this theory for understanding FGM [15, 39]. Similarly, Diffusion of Innovation Theory identifies factors associated with individuals, the innovation, and the environmental and cultural context that are important to the change process. Likewise, the Community Readiness Theory provides a detailed and evidence-based account and practical approach to organisation of change from within the community. Arguably however, although communication between people and reference to individuals is intrinsic to these community-level change theories, they do not offer an explicit consideration of how best to understand individuals or engage with groups of individuals in the context of a particular belief system. 

We argue that integrating community level theories with individualistic theories will provide a framework for understanding how to influence behaviour at an individual and group level in order to facilitate change at a community level. Of the three game-theoretic approaches described above, we would argue that the Community Readiness Model [55] offers the most detailed and practical framework for organising community change, and so we consider this to illustrate integrating community and individualistic approaches. 

### 3.5. How Individualistic (Decision-Theoretic) Models of Behaviour Change Add to Our Understanding

Individualistic models of behaviour change have tended to be used to explain behaviour as performed by a single person (e.g., smoking cessation or reduction of dietary fat intake). The process involved in ending FGM within a community, however, is clearly more complex because it involves cooperation between individuals and families and involves multiple actions and communications by and between multiple actors [47]. In addition, change does not simply occur in a “top-down” manner, but rather change occurs from the “bottom-up” via individuals making particular “choices”. It is also important that individuals who are perceived as “belonging” to FGM affected communities initiate change from the “bottom-up”, as this will improve the level of “buy in” into the proposed change and reduce the possibility of resistance because change will be perceived as occurring from within as opposed to being imposed from the “outside”. Individual level models allow us to gain a better understanding of the range of circumstances that enable or prevent a particular behaviour from occurring. From an individual behaviour change perspective, the elimination of FGM is a goal at the end of a complex chain of behaviour. Therefore, we first need to understand the various behaviours and attitude changes that need to occur at the various links of the chain. Concepts from individualistic social cognitive models and individualistic change models (e.g., self-efficacy, decisional balance, moral norms, risk perceptions, and habitual or emotive behaviour), which often broadly overlap [57, 58], and a developing understanding of how these concepts might translate into effective behaviour change techniques [59] offering mechanisms for understanding how to build messages and activities that are likely to support change within the context of a framework such as the Community Readiness Model.

**Figure 1:**
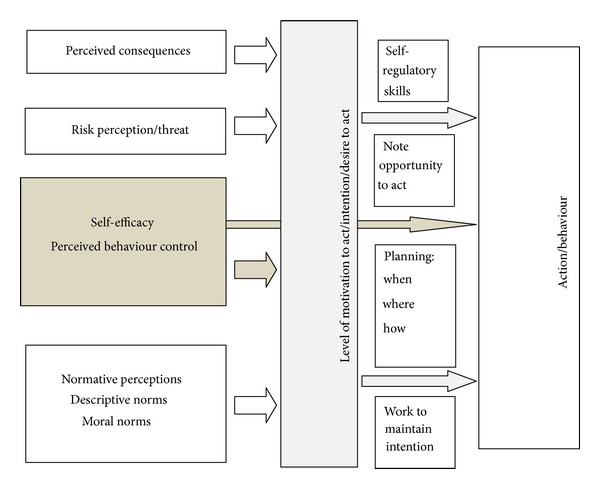
Social Cognitive concepts appearing in many individualistic behaviour change theories.

Figure 1 provides an illustration of the major concepts associated with individualistic behaviour change theories and shows how they are often theorised to relate to action or behaviour.  Table 2 provides a more detailed description of each concept. Please note that, in this context, the action or behaviour we might conceptualise is not necessarily performing or not performing FGM but should include other behaviours that are part of the community change process. For example, in the community readiness approach, general suggestions are offered for activities to support community movement from each stage to the next, and these activities and behaviours can be placed into the behaviour concept depicted in the far right box in Figure 1.

To illustrate further, let us take some of the behavioural suggestions offered by Edwards et al. [55] in Community Readiness Theory to support movement from the *no awareness *stage to the *denial *stage. These include behaviours such as one-on-one visits with community leaders and members and visiting existing and established small groups to inform them of the issue [55]. These sound like common-sense approaches, but the suggestions provide no information about what the content of the communication should or could be. Instead, the onus is on community members to generate common-sense approaches based on their own knowledge and understanding. If we apply Figure 1 in this context, we might use it to achieve two things. First, if we use it to consider beliefs relevant to performing or not performing FGM within the community, it provides us with a framework to gather information about those beliefs and understand something about how to design messages aimed at challenging those beliefs (in a culturally sensitive way and from within the community) that might influence behaviour. Second, if in the process of communicating we are successful in engaging people on the issue of ending FGM, we might use it to consider their beliefs and help them overcome barriers to engaging in one-on-one visits or making one-on-one phone calls.

To further explicate, let us consider a community that is currently at the *no awareness *stage in terms of ending FGM. Perhaps a handful of community members have begun to identify a need for change (i.e., their beliefs have changed such that they no longer favour practising FGM and want their community to change to end the practice.) The community members may already be aware of the types of beliefs their community holds which lead the community to perceive FGM as a favoured practice, but they might want to use the framework depicted in Figure 1 when talking to other community members to further understand and conceptualise the practice. They may also be able to use it to ascertain what changed for themselves to lead them to want to end the practice and conceptualise their own psychological, motivational, and behavioural changes using Figure 1 to help them understand what might work with others. For example, it may be that reevaluating the belief “FGM is required by our religion” was particularly influential for the community members who have already decided they want to bring about change. This belief can be categorised in the *perceived consequences *concept of Figure 1, since an individual who holds this belief is likely to perceive there will be negative spiritual or religious consequences in not performing FGM. Supporting reevaluation of this belief could also be influential therefore amongst others. Where a wide range of beliefs related to the practice of FGM and its continuation are identified, samples of people from the community could become involved in making assessments about the relative importance of some beliefs over others and which beliefs might be most appropriate to target. Thus, they use the framework to design the most appropriate messages to use in the suggested strategies proposed by Community Readiness Theory.

In addition, for those whose beliefs may have changed to favour the idea of ending FGM, Figure 1 may be applied to the behaviours and activities suggested by Community Readiness Theory to progress community change. For example, although an individual or group may be in favour of ending FGM, they may hold beliefs that inhibit their ability to engage in communication with other community members through one-on-one visits or other means. It might be that a female community member lacks the *self-efficacy* (see Figure 1 and Table 2) to talk to a male community leader about the issue, and, whilst it is possible that this may have something to do with that persons intrapersonal capacity to engage in this activity, such as low-self esteem, this inability could just as likely be attributed to, whilst it is possible that this may have something to do with that persons intrapersonal capacity to engage in this activity, such as low-self esteem, this inability could be intrinsically connected to wider social structures relating to gender. Nevertheless, Figure 1 can still be employed to identify this as a constraint, and thus consideration can be given to how individuals acting independently or as part of a group can overcome this barrier in relation to their particular social context. In short we propose that a consideration of psychological or social cognitive factors such as those outlined in Figure 1 could provide a framework for constructing the content of messages and activities required to move people through the stages proposed in Community Readiness theory. 

## 4. Conclusion

In this paper we have argued that there may be utility in integrating community level and individualistic behaviour change theories to ending FGM. However, it is important to note that any intervention programme occurs within a particular context; therefore a “one-size fits all” approach is unlikely to succeed. This is particularly pertinent to the issue of FGM affected communities within the EU, where differing diffusion contexts, such as the length of time individuals and communities, have lived in the EU [46]. The REPLACE project explored the wider sociocultural context of FGM amongst Somali and Sudanese communities living in the Netherlands and the UK. The findings of this community based participatory action research clearly demonstrated that, whilst awareness raising and knowledge are important, particularly with respect to the four traditional approaches to tackling FGM, different communities interpreted and responded to them differently and sometimes in unexpected ways. Many of the campaigners' messages were aimed at the individual and did not take into account the community beliefs which supported the continuation of FGM. This combined with the fact that many campaigns lack a BCT basis has resulted in slow progress in ending FGM in the EU. 

REPLACE demonstrated that all intervention efforts should begin with a process of community based participatory action research and/or exploration of the current belief systems relevant to any given community before conducting any behaviour change. This is supported by Glanz [60] who posits that participatory action research methods are an integral approach to intervention and evaluation in communities. Furthermore, these methods are consistent with group decision making and allowing the community to take ownership of the change strategies. What we suggest is that, where community-based action is taken in collaboration with those interested in application of behavioural change approaches, an approach that combines community and individualistic approaches is fostered. Clearly, taking such an approach is likely to be time and resource intensive, but we would argue that because the practice is complex, and the time and intensity of fully understanding the nature of continuation are required in order to begin to understand what might work best to end the practice in a given community. 

The proposed integration of behavioural change theoretical ideas that we have outlined in this paper is intended to extend the debate and contribute to understandings of how behavioural change approaches might be applied to the issue of ending FGM. We do not assert that this is the only way to consider behaviour change approaches in relation to this issue, and we recommend that more empirical and evaluative work is undertaken to assess the validity and utility of such an approach. Indeed, the work produced by Michie et al. [58] provides a potentially valuable insight by placing behaviour within particular contexts and seeing behaviour and interventions as part of a “system”, in which an intervention may have a consequence for other parts of the “system”, which might work against sustainable change or in favour of it. This is particularly important in relation to FGM, with particular messages, such as those associated with health operating at different levels and being interconnected with religious beliefs [46]. Furthermore, Michie et al.'s [58] approach acknowledges the complexity associated with agency, that individuals are not a disembodied reason, but act in the way they do because of habit or emotional and social reward. Indeed, we would argue that interventions need to seriously address the emotive and social aspects associated with motivation in relation to FGM and not simply appeal to individuals' reason. Finally, interventions need to be multidimensional and focus on individual, community, and societal level change. This not only demands a multiagency response in terms of third sector and public sector services, but it requires a multidisciplinary participatory approach in order to construct a sound theoretical basis and evaluation of behaviour change approaches to FGM. 

## Figures and Tables

**Table 1:** Stages of community readiness model (adapted from Edwards et al. [55]).

Stage	Description
No awareness	(i) Community members not conscious of the problem. (ii) Accepting of the issue as part of the way things are.
Denial	(i) Some awareness amongst some community members. (ii) No motivation to act or belief that anything can be done.
Vague awareness	(i) Some community members communicate in general terms about problem. (ii) Poor understanding and no motivation change things.
Preplanning	(i) Clear recognition of the problem. (ii) Community leaders are motivated to take action. (iii) No clear understanding about what action to take.
Preparation	(i) Planning begins to take on focus and detail. (ii) Data may be formally collected to use in planning. (iii) Decisions are made about what needs to be done. (iv) Resources are gathered and put to use. (v) Some community support.
Initiation	(i) Activity or action may have started but is perceived as novel. (ii) Leaders enthusiastic. (iii) Community support.
Stabilisation	(i) General support remains. (ii) Some prevalence tracking going on, supported by an organised and experienced administration. (iii) Ongoing evaluation of efforts likely and low motivation for change or progression.
Confirmation/expansion	(i) Support has grown, and authorities and policy-makers are likely to be on board. (ii) Some evaluation is likely to have happened. (iii) New efforts initiated with plans to reach new and difficult to access groups.
Professionalization	(i) Knowledge and understanding of problem is sophisticated. (ii) Administration is highly skilled. (iii) Community involvement is high, and ongoing evaluation and adaptation are typical.

**Table 2:** Explanation of the major concepts from individualistic behaviour change theories (BCTs) adapted from Conner and Norman [57].

Perceived consequences	An individual might hold any number of relevant and salient beliefs or perceptions about the consequences or outcomes of performing (or not performing) a given behaviour (e.g., “having my daughter cut will protect her virginity” or “not having my daughter cut will lead to her being ostracised by our community”). They will also hold evaluations about how desirable such an outcome is (e.g., “protecting my daughter's virginity is extremely important and desirable”).

Risk perception/perceived threat	This is often assessed as measures of perceived susceptibility/vulnerability and perceived severity of the threat. Some have argued that this element of BCTs could be subsumed by *perceived consequences*, in that one might perceive the susceptibility of a threat as an outcome belief (e.g., “going ahead with my daughter's circumcision may cause her to have pain and infections”), and an evaluation of that outcome is akin to perceived severity (e.g., “my daughter experiencing pain and infection is a very bad thing”).

Self-efficacy and perceived behavioural control (PBC)	Self-efficacy is often described as confidence in one's ability to perform a particular behaviour, and PBC as perceptions about how in control of performing a behaviour one is. This concept can be thought of as perceptions about barriers or facilitators to action, and these can be both perceived or real; for example, a person may perceive that there is a barrier preventing them from carrying out an action, but in some cases there might actually be real, tangible barriers, such as not having the power or resources to carry out a certain behaviour. Barriers and facilitators can also relate to an individuals' internal (skill-based) and external factors. Believing that one is able to perform a behaviour is critical to the likelihood of performance (e.g., “I know the right people and have enough money to arrange for my daughter to be circumcised” or “I have the strength of character and the conviction to defend my decision not to circumcise my daughter”).

Link between self-efficacy/PBC and behaviour	Two theories, the theory of planed behaviour (TPB) and social cognitive theory (SCT), propose a direct relationship between self-efficacy or perceived control behaviour (PBC) and behaviour, as well as a relationship with intention or motivation to act. This is illustrated along with other concepts in Figure 1. Essentially, what this demonstrates is that where perceptions about ability to perform a behaviour reflect actual abilities, there will be a direct impact on behaviour regardless of how motivated an individual is. Ajzen, [48]; e.g., a mother may want to prevent her daughter from being cut but may lack control over this and fail to prevent the cutting.

Normative influences	These are included in various ways in BCTs, and discussion of their involvement in behavioural change has been prevalent. Normative influences refer to perceptions one has about what important individuals others think you should do with regards to a given behaviour (normative beliefs), perceptions about what other people do themselves (descriptive norm), and beliefs about what is right (moral norms). It seems likely that normative influences are very strongly related to decision-making related to FGM.

Intention	This is also known as motivation or desire to carry out a particular action. Intention mediates the relationship between social cognitive processes outlined thus far and behaviour. The only exception is self-efficacy/perceived behaviour control, which, as already outlined, can also have a direct impact on behaviour. The first three stages of change (from the TTM), outlined and adapted in Shell-Duncan and colleagues work on FGM [39], can be said to represent a measure, or continuum, of increasing intention or motivation.

Self-regulatory skills	Once a decision or intention to act has been made, there is consensus by theorists and researchers about the processes involved in translating intention into action. It is widely held that, in order to maintain and remember intentions and respond to opportunities when they arise, various self-regulatory skills are required. Intentions need to be strengthened and protected once formed, and careful plans about how, when, and where are required.

Behaviour or action	This might refer to any behaviour or action relevant to performing or not performing FGM and might include communication about FGM with other community members or any behaviour or activity suggested within Community Readiness Theory.
